# Get Outside! Promoting Adolescent Health through Outdoor After-School Activity

**DOI:** 10.3390/ijerph18147223

**Published:** 2021-07-06

**Authors:** Patricia Ann Barfield, Katelyn Ridder, Justin Hughes, Kelly Rice-McNeil

**Affiliations:** 1 School of Nursing, Oregon Health & Science University (OHSU), Portland, OR 97239, USA; 2 Health & Human Performance, Eastern Oregon University, La Grande, OR 97850, USA; kperickson@eou.edu (K.R.); jhughes@eou.edu (J.H.); krice@eou.edu (K.R.-M.)

**Keywords:** adolescent, health promotion, outdoor activity, qualitative inquiry, rural

## Abstract

Background: The Get Outside: After School Activity Program (GO-ASAP) exemplifies how a rural community can utilize its natural resources and community partnerships to promote adolescent health. Methods: A qualitative descriptive inquiry was conducted using convenience sampling. Data were collected from students (*n* = 13/2018; *n* = 13/2019) via focus group and art-based method (2018 only) and parent (*n* = 6/2018) focus group. Data were analyzed via qualitative content analysis using the applied theoretical frameworks of Social Cognitive Theory and Social Determination Theory. Results: (1) Increasing Health-Related Competencies. Students increased their physical activity, improved their sleep, perceived less stress, and reported changes in dietary habits and electronic use. (2) Increasing Social Relatedness. Students made new friends, felt more connected, and spent less time home alone after school. (3) Increasing Autonomy and Intrinsic Motivation. Students recognized their emerging capabilities, and their increased confidence stimulated more action-oriented behavior. Parent-perceived changes support and mirror student reports. Conclusion: Outdoor, nature-based, activity programs are a novel upstream approach to promote adolescent health, especially in rural communities where natural resources often exceed health-service resources and community partnerships are a way of life.

## 1. Introduction

Physical and mental health problems are on the rise in younger generations [1]. Many of the underlying health risks are rooted in detrimental health-related behaviors that begin in childhood and emerge as lifestyle choices during adolescence [2,3]. Rural-dwelling adolescents may have a greater burden of health risks compared to their urban counterparts, in part due to differences in health behaviors, economic disparities, and a lack of access to health-promoting services [4,5]. The onset of physical and mental health problems in adolescence can disrupt typical development and extend health risks into adulthood [6]. Establishing early, health-promoting behaviors is vital to mitigating health risks across the lifespan. Adolescent health-promotion targets often include health-related behaviors such as physical activity, sleep, dietary choice, and limiting electronic use [7,8]. Yet the data reveals that adolescent health-promoting behaviors have not improved greatly in the past 10 years [9].

A national survey of U.S. high school students (*n* = 14,765) indicates that adolescents are not meeting the recommended health promotion and disease prevention goals [2]. Less than half (46.5%) get 60 min of exercise 5 or more days per week, one-quarter (25.4%) receive the recommended 8-h or more of sleep each night, 43% use electronics 3 or more hours per day, far exceeding the recommended 1-h per day recommendation, and nearly 20% (18.7%) consume 1 or more soft drinks daily [2]. Of the adolescents surveyed, 31% reported feeling sad for 2 weeks or more in the past year, and 17% endorsed thoughts of suicide [2]. Moreover, adolescent health-promoting behaviors are a global concern. In a pooled analysis of adolescent (*n* = 1.6 million) survey data taken from 298 schools across 146 countries, 81% were reported deficient in health-promoting physical activity [10]. There is a clear need to explore novel holistic health-promoting interventions to effect positive change in adolescent physical and mental health.

A growing approach to adolescent health promotion and disease prevention is participation in outdoor, nature-based, or “green” activities [11]. Although outdoor activity programs have been used for educational [12], therapeutic [13], and focused health targets (e.g., diabetes, asthma; [14]) the physical and mental health promotion and disease prevention benefits of outdoor, nature-based, or “green” activity programs for adolescents are promising but only now emerging [15,16]. The authors of one systematic review who extracted data from 84 publications, reported a 60% improvement across all *(n =* 56) health outcomes—physical, mental, and social, in children and adolescents participating in a nature-immersed experience [17]. Their evidence suggests that when adolescents are outdoors in nature they are more likely to engage in health-promoting behaviors. Outdoor activity is associated with an increase in adolescent physical activity [18,19], especially when engaged in a preferred activity [11]. In rural adolescents, outdoor time has been associated with reduced electronic screen time [20]. Adolescents participating in outdoor activity report a decrease in psychological symptoms (e.g., depression, irritability, anxiety) and somatic distress (e.g., headache, stomachache; [21]). There is recent evidence suggesting outdoor activity may help adolescents to feel more socially connected [22]. In short, the implementation of outdoor, nature-based, or “green” health-promoting activities might best be viewed as a novel upstream approach to improve adolescent health-promoting behavior.

The purpose of this article is to share the adolescent and parent perspective from a qualitative inquiry that sought to understand the influence of a rural outdoor afterschool program on adolescent activity and health-related behaviors. The Get Outside: After School Activity Program (GO-ASAP) serves as an exemplar.

### The Get Outside: After School Activity Program (GO-ASAP)

GO-ASAP is an outdoor afterschool program developed and delivered by faculty and students from Eastern Oregon University (EOU), designated ‘Oregon’s Rural University”. The primary thrust of GO-ASAP is to increase physical activity by introducing a variety of outdoor noncompetitive activities to promote healthy behaviors and reduce health risks. The program leverages the natural rural resources, including the Blue Mountains to the west, the Wallowa Mountains to the east, and the Grande Ronde River. GO-ASAP offers outdoor activities and health education sessions twice weekly in 90–180 min sessions in two 10-week blocks (Table 1). Other activities include several extended weekend outings and a multi-day backpacking trip. Community partners lend program support, outdoor experts provide requisite expertise and training, outdoor gear vendors provide equipment (e.g., bikes, paddleboards, kayaks), and the city parks and recreation department supplies vehicles for transportation. Financial support has been provided through grants (e.g., American Cancer Society) and community-based organizations, including local hospital and public health systems.

A particular strength of the GO-ASAP program is the active participation of undergraduate and graduate university students with varied majors/minors (e.g., Exercise Science, Education, Community Health, Outdoor Recreation, and Nursing). All university students obtain valuable hands-on experience within their respective fields by helping to assess adolescent health, plan and deliver educational and outdoor activity, and evaluate the effectiveness of their interventions. In addition, the university students lend vital support to the adolescent participants via role modeling and mentoring.

The GO-ASAP program is framed by two motivational theories, Self Determination Theory (SDT) [23], and Social Cognitive Theory (SCT) [24,25]. SDT helps to explain how intrinsic motivation is facilitated by fostering a sense of relatedness, competence, and autonomy through demonstrations of respect, caring, inclusivity, and the provision of positive feedback and choice [23,26]. SCT emphasizes the interactive relationships among person, environment, and behavior to cultivate competence, self-efficacy, and motivation [24]. Competence, operationalized as developing knowledge and skill, improves through role modeling, practice, personal experience, and positive (social and environmental) feedback. The interaction among emerging competence, self-efficacy, and intrinsic motivation activates health-promoting behavior such as physical activity [26]. These two theoretical frames lend strong conceptual support for adolescents’ achievement of the overall program goals: To increase health-related competencies, social relatedness, autonomy, and intrinsic motivation, through health education and outdoor activity. Program activities/interventions are designed to support the theoretical constructs. A GO-ASAP Intervention Manual was created, and all team leaders were trained on the contents and specific motivational theories.

## 2. Materials and Methods

We employed a qualitative descriptive design [27]. A convenience sample of two student cohorts (2018/2019) and one parent cohort (2018) from the GO-ASAP program were included. Students in the 7th and 8th grade are eligible for program enrollment and retain their eligibility through high school. Exclusion criteria include participation in organized sports or extracurricular activities, as the program goal is to increase the physical activity and health behaviors of inactive students. Students in this sample were recruited from one rural school district via indirect (e.g., letters) and direct (e.g., face-to-face) methods. First, the school principal and counselor identified eligible students (e.g., those not involved in organized sports or extracurricular activities). Next, the school sent a letter to parents of eligible students providing introductory program information. Parents returned the letter to the school indicating their child’s interest in participating in the program. The GO-ASAP team members followed up with interested students/parents to provide further program information, answer questions, and conduct enrollment.

### 2.1. Ethical Considerations

Institutional Review Board approval was obtained from the participating university. Prior to data collection, students provided assent, and their parents’ provided informed consent for their own and student participation. All participation was voluntary, and students maintained the right to withdraw from participation at any time.

### 2.2. Data Collection

Data were collected in separate one-time student (2018/2019) and parent (2018) focus groups using a semi-structured interview guide (Appendix A Table A1). This approach aligns with the theoretical drive to promote autonomy; it encourages participant exchange (e.g., peer to peer/parent to parent), and affords the interviewer(s) opportunity to make clarifications and conduct member checks in real time. In some instances, questions were direct and involved students raising their hands for a numerical count to gauge the collective response (e.g., raise your hand if GO-ASAP has affected your sleep). In 2018, students were engaged in a single art-based *Draw-and-Tell Conversation* (DTC) [28] activity to elicit a more personalized perspective of the program influence. Students were provided with art supplies, and prompted to think about their life before and after GO-ASAP. Next, they were asked to draw and tell about their program experience. Drawing and then telling about their experiences afforded the students a more reflective response.

### 2.3. Data Analysis

Focus group data were analyzed via qualitative content analysis. This inductive approach includes coding, categorizing, and organizing data into thematic lines in order to present a straightforward account of experience [29]. Audio recordings were transcribed verbatim. All transcripts were read line-by-line in an iterative fashion, and text excerpts pertinent to the interview questions, probes and theoretical constructs (e.g., autonomy, relatedness) were highlighted. Text excerpts were given *data-near* codes and then compared within/across cases to identify similarities, differences, and connections [30]. Ongoing analysis was supported by the creation of data matrices, organized by theoretical and thematic lines, and supported by direct quotations. Data from the DTC were analyzed similarly, focusing on the students’ accompanying narratives. Although the drawings were examined for content, contrast, and described meaning, no interpretations of drawings were made. Rigor and descriptive validity were supported by, (a) prolonged engagement of an experienced qualitative researcher (first author) with the data, (b) research peer review debriefs with an external PhD prepared qualitative research expert, and (c) triangulation of data from different perspectives (e.g., student/parent), and different methods (e.g., focus group/DTC) [27,31].

## 3. Results

A total of 26 students participated in the 2018/2019 GO-ASAP program (*n =* 13 in 2018; *n =* 13 in 2019). The majority (58%) of participants were enrolled in middle school. Student demographics are displayed in Table 2. The 2018 parent (*n =* 6) focus group data are included to broaden the contextual lens of the GO-ASAP program, referred to as “the program” going forward. No parent demographic data were collected. The average duration for focus groups was 34 min.

The students’ responses within and across the data, reflected an overwhelming presence of *I-statements* and the use of *active* vs. passive voice. Equally notable were their reflections of engagement in program activities and internal vs. external rewards. The students reflected on their perceived changes and developing competencies (e.g., knowledge and skill), through relationships with others, and the discovery of their own inner strength. Their reflections were often mirrored in parent comments. The students’ *I-statements* provided the key analytical structure, as thematically they aligned with the theoretical underpinnings (i.e., SDT, SCT) and program goals (Table 3). As this study intentionally privileged the adolescent voice, the student results are presented first within the frame of the program goals, with parent results following.

### 3.1. Increasing Health-Related Competencies

Students described positive changes in their health-related behaviors that were reflective of developing health-related competencies. The catalyst to change was to get them outside. Most students (61%) explicitly described moving from the inside to the outside because of program participation. Once outside, students described being more physically active, using their electronics less, sleeping better, and feeling less stressed.

#### 3.1.1. “Now I Do More”

**Figure 1:**
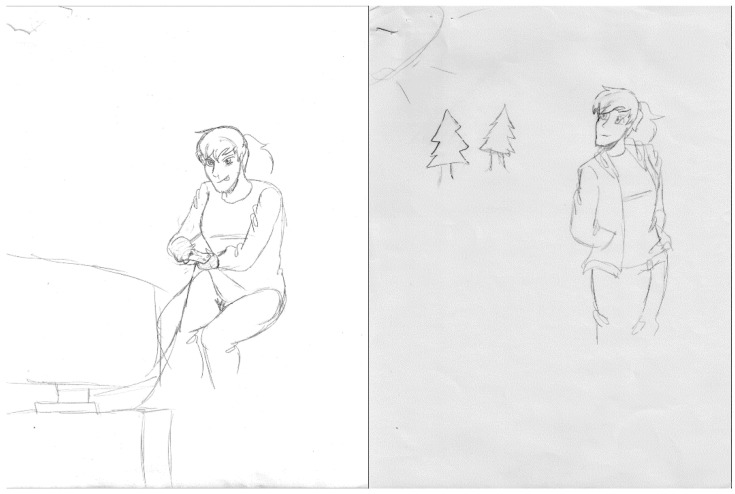
Getting Outside.

Before participating in the program, students described going home after school where they would, “just stay inside” (S3) or “play video games” (S10). In some instances the before and after program changes were quite striking “[Before] I didn’t get outside much… I didn’t really like the outdoors… [After] Now I’m like almost always outdoors” (S1). Students increased their physical activity once outside, even on non-program days. Some students even tried to persuade their parents to join them, “I’m more active… I’ve been trying to convince my parents to do more outdoor stuff with me...” (S10). Outdoor physical activities included going for walks, bike riding, hiking, and playing soccer. Being outside and active meant students were spending less time on their electronic devices. This change is depicted in one student’s DTC story (Figure 1) that depicts her going from being engrossed in playing video games inside to being outside in the forest.

#### 3.1.2. “I Sleep Better”

Students reported getting more sleep and sleeping better. Even though the majority of students (85%) agreed they were sleeping better, they did not change their bedtime or perceive much change in their sleep habits. This perspective is exemplified by one student’s comment, “I don’t really think it’s changed my sleeping habits that much, but I do sleep a lot better now” (S17). Students did, however, associate changes in their physical activity levels with improved sleep, “I’m getting physical activity every day and that helps me sleep so much more” (S20). They also reported positive changes in their diets, such as avoiding caffeine at night and making healthier diet choices that could have contributed to better quality of sleep.

#### 3.1.3. “I Feel Less Stressed”

For half of the students (50%), just being outside eliminated or alleviated their stress, “Outside there’s no stress” (S22). Other students expressed feeling better prepared to cope with stress or better able to manage the feelings of stress. For example, one student described feeling irritable when stressed, “before I used to punch walls… threaten people and stuff like that, but now I’ve gotten out in nature more and that really helps” (S24). Similarly, participating in an outdoor afterschool activity program gave students a break from the usual pressures of school, helping them to “leave all of the stuff from school behind” (S21) in a way that going home after school did not.

### 3.2. Increasing Social Relatedness

The student-perceived changes in social relations were discussed in animated fashion—students laughed and joked with each other as they talked about their newly formed friendships and shared experiences. However, they described the changes in their social relatedness in stark contrast of going from nothing to something. Participating in the program gave students an opportunity for socialization through shared activity they did not previously have.

#### “I Am Not Alone”

**Figure 2:**
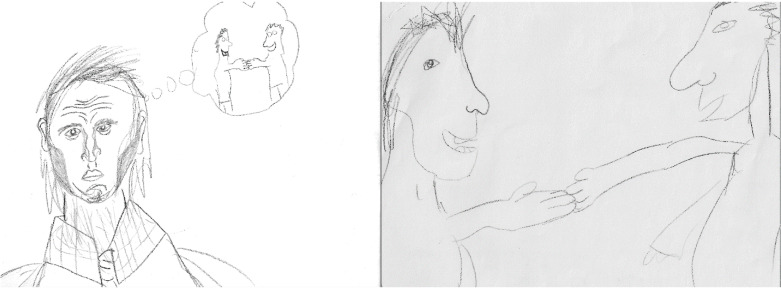
Making a Friend.

Nearly half (42%) of the students explained that since participating in the program, they went from being home alone in their rooms after school, to “hanging out with friends” (S7) after school. One student summarized the change echoed by peers as going from “not having friends or doing very much” to “meeting people and doing stuff” (S14). Having friends to do things with made their lives “more fun” (S20), helped them to “try new things” (22), and widened their circle of trust—“... now there’s a bunch of people I can trust” (S10). In some instances, the opportunity to make more friends increased a student’s interest in being social. For example, one student explained, “Before I preferred to be alone, but this changed me wanting to be alone” (S10). Away from the confines of their bedrooms, students spent less time alone and lonely. This was noted in absolute terms, “I am not alone” (S8) and in more nuanced terms, “I am still lonely but better” (S13). The accompanying DTC story (Figure 2) depicts one student moving from thinking about making a friend to actually making a friend.

### 3.3. Increasing Autonomy and Intrinsic Motivation

It is difficult to separate the students’ described sense of growing autonomy from their emerging intrinsic motivation. Students recognized the changes in their own self-perception and abilities and discussed them as antecedent to their increased interest and intent to be more active. Much of the self-realization preceding action was in the context of outdoor physical activity and accomplishment. Students did things (e.g., hiking, rock climbing, kayaking) they did not believe themselves capable of doing, but given the opportunity, support, and reinforcement, they realized they were more capable than they thought—this stimulated their intrinsic motivation.

#### “I Am Stronger Than I Thought”

**Figure 3:**
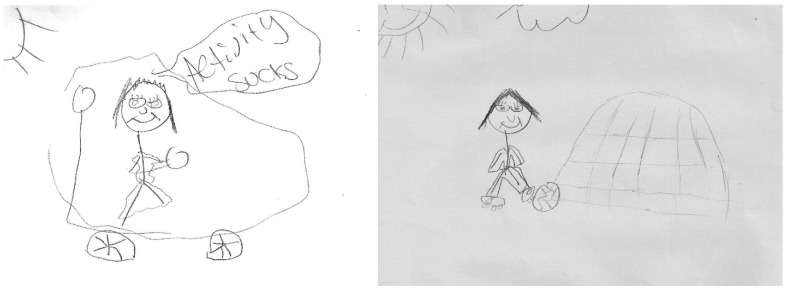
More physically active.

Participation in the program changed the way students described themselves and their motivation to be active. Students remarked that before participating in the program they were, “self-conscious” (S12), “lazy” (S7), “bored, not motivated” (S8). One student summarized the group’s remarks, “like pretty much everyone else, I would just stay in my room most the time and just do whatever” (S12). However, after participating in the program students expressed feeling more self-assured and independent—“I am stronger than I thought” (S25). Thus, they moved from feeling “self-conscious” and inhibited (away from action) to “confident and happy” (S12) (towards action) and were more motivated to be physically active—“now I am happy to exercise… very excited to be active” (S7). This transformation is evident in a DTC story (Figure 3) about always riding in a car thinking “activity sucks” to a change of wanting to be outside and playing soccer (S3).

### 3.4. Parent-Perceived Changes

#### “A Whole New Child”

All parents agreed their teen’s interest in being outside and physically active had increased with program participation. One parent explained that before her son was enrolled in the program he would “sit on the couch and watch a movie all day long… Now he comes home and he is like mom, we got to get better!” (P2). Similarly, another parent shared, “My son use to sit around playing video games all day long and now he always wants to leave, go riding his bike” (P4). Parents also noted a change in their teen’s interest in improving their diet—“He used to be that junk food kid. Now he comes home and he’s talking about healthy choices. I haven’t seen him eat a bag of potato chips in weeks. He won’t eat junk food, won’t even drink soda… literally everything fresh and good for him” (P3).

In regards to social relatedness, parents discussed the observed difference in terms of “fit” or “fitting-in” (P3). For example, one mother explained that her daughter had never “fit in” at school because she did not play sports or belong to any club. The program changed this—“Now, she is part of something [you know] where she doesn’t fit in anywhere else, like with team sports or club stuff…. It doesn’t matter if you’re really good at it or if you’re not so good at it… You still fit it in” (P5).

For some parents, the observed changes in their teen were dramatic. “He’s totally bloomed… I see a whole new child” (P3). Students went from not wanting to do anything to wanting to be involved. Like the adolescents, parents commented on their teen’s growing sense of self-confidence and competence that led to increased autonomy and intrinsic motivation. As one father shared, the emotion evident in his voice, “For my boy… it was just confidence... He was more able to do things by himself without having to second-guess himself or asking... he was more just do it” (P2). The father went on to explain that before the program he had tried to get his son to participate in events, sports and such, but that he would just “sit there, he wouldn’t even get out of the car... now he’s like... I’m doing this!”

## 4. Discussion

The GO-ASAP program exemplifies the potential for rural community collaboration to increase physical activity, promote healthy behaviors, and reduce health risks in adolescents in the present and going forward. The strong theoretical underpinning lends critical infrastructure to the program design, intervention(s), and evaluation. Evident across all the data is the interplay between theoretical constructs, explicitly personal, relational, and environmental factors (i.e., SCT) that helped to increase student health-related competencies, social relatedness, autonomy, and intrinsic motivation (i.e., SDT). Woven within the program delivery are the theoretical threads of respect, caring, support, role modeling, and positive feedback. This novel, upstream, rural-based program is part of a growing movement focused on increasing outdoor, nature-based, or “green” activities for health promotion and disease prevention [11,17,19]. Engaging adolescents in outdoor health-promoting activity is a strategy that can be implemented specific to the space and regional resources of any community or country.

The students who participated in GO-ASAP were more physically active, even on non-program days. This finding was endorsed by students and parents, and fits well within the literature that affirms adolescents are less sedentary and more physically active when outdoors compared to indoors [18,32]. The program leverages motivational and developmental knowledge, offering outdoor experiences that are fun, challenging, and that offer a level of personal preference. The combination of stimulating activity and preferred choice was essential to engaging adolescents, who by their developmental stage seek novelty and independence [3]. Adolescents who are engaged in preferred outdoor physical activity are more likely to meet the minimum standards for vigorous physical activity and to achieve higher levels of well-being [11]. The GO-ASAP students were more physically active and more intrinsically motivated to be active. Parents witnessed their children self-propel from being indoors and inactive, to being outdoors and active. In the process, students developed a sense of pride in their newly developed competencies and a recognition of their inner strengths.

In addition to being more physically active, students endorsed getting more (and better) sleep, using electronics less, and making positive dietary changes (e.g., limiting caffeine at night), the latter two may also have helped to improve their sleep. Sleep is particularly problematic for adolescents, for reasons Carskadon [33] labeled as “the perfect storm” of bioregulatory, psychosocial, and societal pressures placed upon them (p. 637). The bidirectional relationship between exercise and sleep is well accepted, and the health benefits of sleep are well understood [34]; however, the science of outdoor physical activity and sleep is still under scrutiny, especially for adolescents [19]. In contrast, excessive electronic use is a known disruptor of adolescent outdoor time [20], physical activity [18], and sleep [35].

It is unknown how much of the positive behavioral changes and health competencies are attributable to GO-ASAP’s individual components, for example, health education, outdoor physical activity, staff mentoring, role modeling, or other “personal” or environmental factors. The findings, however, fit within the frame that highlights the many pathways outdoor, nature-based activity can positively influence health and health-related behavior [36,37]. The ability to promote adolescent health and prevent disease through outdoor activity has serious health, economic, and policy implications, especially in rural communities where the burden of chronic disease is higher and the access to health resources is limited.

One of the most interesting findings centered on these teens’ stress experience. For some, being immersed in nature was stress relieving; for others, the outdoor after-school activity provided respite and relief from school stress, and/or a fun alternative to being home alone. The awareness that outdoor, nature-based activities offer relief and restoration from stress is not new [38], and the greatest benefit is said to occur when individuals feel a sense of escaping stressors and/or stress-inducing situations [39]. Students participating in GO-ASAP experienced a buffering of their school stress. Researchers have documented statistical association of outdoor physical activity or “green exercise” with reduced stress, renewal, and enhanced health and well-being [37,40,41], but the modes of measurement have varied such that no clear signal emerges. Chronic stress is antecedent to chronic disease, poor physical and mental health outcomes, and health-harming behaviors [42,43]. Adolescents have a heightened sensitivity and response to stress due to neurochemical processes and changes; however, equally important is their enhanced capacity to develop resilience due to brain neuroplasticity [3]. Outdoor nature-based activities may not only help to reduce adolescent vulnerabilities to stress, but also increase their ability to manage stress in the present and going forward.

The adolescent world centers on social relationships. The GO-ASAP program experience expanded the students’ social circle and perceived social support. The students became more socially connected; they made friends and felt less alone. From a parental perspective, students found a place to “fit in.” This finding, while not surprising because the program engages students not involved in organized school-related activities, has weighty implications. Investigations into outdoor nature-based programs/interventions often probe psychological symptoms, emotional well-being, self-concepts, and health-risk behavior [19,21,44]. Fewer inquiries examine the impact on social relationships, and yet social relations are essential to healthy adolescent development [19]. In the context of adolescent suicidality, social relatedness—those connections to family, friends, and school— are vital to reducing the risk of suicide [45]. In a nation where adolescent suicide rates continue to climb, outdoor afterschool nature-based programs may have a role in increasing the protective relational factors critical to reducing suicide risks [46].

Increasing autonomy is a hallmark of typical adolescent development [3]. As adolescents grow in knowledge, skill, and ability, they become more competent, confident, autonomous, and intrinsically motivated [23,26]. Students in the GO-ASAP program gained in their health-related competencies related to physical activity, sleep, diet, and social interactions. For some students, the growth appeared to be more dramatic, suggesting that for some inhibited and/or amotivated rural teens, an outdoor afterschool program may be particularly impactful.

### Limitations and Recommendations

A single time point of inquiry and the inability to create direct dyadic alignment between the individual students and their parent limits this study. The ability to compare all data points (student, DTC/parent) across time (2018/2019) would have extended the contextual lens of how a rural outdoor afterschool program influences adolescent activity and health-related behavior. Lastly, generalizability and transferability of the findings are limited to parallel rural populations with similar characteristics, amenities and resources.

Recommendations for future research include continued investigation into how outdoor after-school activity programs might improve adolescent health and health-related behaviors to mitigate targeted disease-risk factors (e.g., physical inactivity, sleep, diet) across different populations, especially culturally diverse adolescents. Also warranted, from a clinical perspective, is the use of outdoor after-school activity programs to improve holistic health outcomes for teens with known physical and mental health conditions.

## 5. Conclusions

The onset of physical and mental health problems during adolescence can disrupt normative development and create a cascade of damaging health-related risks that extend into adulthood. Many of the health-related behaviors that increase or decrease risk of developing physical or mental health problems takes root during adolescence. Outdoor nature-based activity represents one upstream approach to health promotion and disease prevention, and provides knowledge-based and experiential activities that foster adolescent physical, mental, and social growth. Such programs may be even more important in rural communities and underdeveloped countries where poverty and chronic disease exceed available health-promoting resources. The GO-ASAP program is an exemplar of how a rural community can promote adolescent health through outdoor activity.

## Figures and Tables

**Table 1:** Examples of Activities and Health Topics.

Activities	Health Topics
Skiing	Nutrition
Snowboarding	Components of Health
Rock Climbing	Stress
Yoga	Sleep
Mountain Biking	Resilience
Hiking	Effects of Alcohol & Drugs
Paddle Board/Kayaking	Blood Pressure

**Table 2:** 2018–2019 Student (*n* = 26) Demographics.

**Female**	50%
**Mean Age**	14 years
**7th Grade**	27%
**8th Grade**	31%
**9th Grade**	31%
**10th Grade**	11%
**White**	75%
**American Indian/Alaska Native**	7%
**Native Hawaiian/Pacific Islander**	8%
**Hispanic/Latino**	4%
**White/Hispanic**	4%
**Black/African American**	3%

**Table 3:** Student Focus Group Data.

PROGRAM GOALS	STUDENT I-STATEMENTS
**INCREASE HEALTH-RELATED COMPETENCIES**	“I do more.” (S3) “I sleep better.” (S17) “I feel less stressed.” (S25)
**INCREASE SOCIAL RELATEDNESS**	“I have made new friends.” (S4) “I am not alone.” (S8)
**INCREASE AUTONOMY**	“I am stronger than I thought.” (S25) “I am less self-conscious and more confident.” (S12)
**INCREASE INTRINSIC MOTIVATION**	“I wouldn’t do anything when I got home, I’d just stay inside and not do anything, but now... I like to do stuff outside of school.” (S3)

## Data Availability

Data supporting reported results can be accessed by contacting the authors.

## Appendix A

**Table A1:** Focus Group Semi-Structured Interview Guide(s).

Student Interview Guide 2018 (*n* = 13)	Student Interview Guide 2019 (*n* = 13)	Parent Interview Guide 2018 (*n* = 6)
Has GO-ASAP affected your life? If so, how? Has GO-ASAP affected your physical activity? If so how? Has GO-ASAP affected your friendships? If so how? How do you feel about the GO-ASAP activities? How has the GO-ASAP activities affected your health if at all? Are there any accomplishments from this year that you’d like to share?	Has GO-ASAP influenced your life? If so, how? Has GO-ASAP affected your stress levels? If so, how? Has GO-ASAP influenced your sleep habits? If so, how? What was your favorite thing about GO-ASAP? Has GO-ASAP affected your health? If so, how? Are there any accomplishments from this year that you’d like to share?	What did you like best about your child participating in the program? What changes have you seen in your child from the start of GO-ASAP until now? Do you think GO-ASAP had an impact on your child’s physical activity behaviors? How? Do you think your family’s physical activity behaviors have changed because of your child’s participation in this program? How? If we did this again next year, would you want your child to come back?
